# Design and Calibration of a Slit Light Source for Infrared Deflectometry

**DOI:** 10.3390/s25030944

**Published:** 2025-02-05

**Authors:** Lu Ye, Xiangchao Zhang, Min Xu, Wei Wang

**Affiliations:** 1 Shanghai Engineering Research Center of Ultra-Precision Optical Manufacturing, School of Information Science and Technology, Fudan University, Shanghai 200438, China; 19110720064@fudan.edu.cn (L.Y.); zxchao@fudan.edu.cn (X.Z.); minx@fudan.edu.cn (M.X.); 2 Shanghai Institute of Technical Physics, Chinese Academy of Sciences, Shanghai 200083, China

**Keywords:** optical measurement, infrared deflectometry, aspherical surface, grinding, camera calibration

## Abstract

Infrared deflectometry is an efficient and accurate measuring method for curved surfaces fabricated via grinding or finish milling. The emitting properties and geometrical configurations of the infrared light source is a core component governing the measurement performance. In this paper, an infrared slit light source is designed based on the cavity structure of a polyimide heating film. This design ensures good stability and uniformity of the light source whilst effectively reducing background noise. Additionally, the light source can be applied as a calibration board for calibrating infrared cameras. The light source is aligned using a theodolite and cubic prism to control the positional deviations during scanning. Experimental results demonstrate that the proposed slit light source and calibration method can achieve a measurement accuracy of 1 µm RMS, which can meet the needs of rapid measurement in grinding. This approach provides a reliable, cost-effective, and efficient tool for surface quality assessments in optical workshops and has a broad application potential.

## 1. Introduction

With the advancement of optical system resolution, there is a growing demand for large-aperture, complex curved mirrors in various fields such as astronomical observation and national defense [1,2,3,4,5]. Measuring these mirrors presents significant challenges and is often time-consuming. During the grinding stage, the deviation between the actual and ideal surface shapes is substantial, typically ranging from 0.01 mm to 1 mm. The primary goal at this stage is to quickly enhance surface accuracy and reduce surface roughness while minimizing the workload of subsequent polishing processes. Traditional measurement methods, such as coordinate measuring machines (CMMs) and laser trackers [6,7], have notable drawbacks, including limited accuracy, long measurement time, high cost, and restrictions on mirror size due to the structural limitations of the instruments. To address these issues, non-contact measurement techniques, such as infrared interferometry, have been developed. However, infrared interferometry requires specific infrared null optics or computer-generated holograms tailored to each aspherical surface shape [8,9]. These components are expensive, difficult to fabricate, and challenging to align, further complicating the measurement process. To overcome these limitations, infrared deflectometry (IRDM) has been developed to measure aspherical surfaces during the grinding stage. IRDM offers several advantages, including a large dynamic range, high measurement speed, and low hardware costs. It provides a viable alternative to costly CMMs and laser trackers, enabling shorter measurement cycles and improved efficiency.

A key challenge in IRDM is the lack of a suitable technique to generate dynamic thermal fringe patterns in the long-wave infrared range [10,11,12,13,14]. Su et al. [15,16] proposed the use of a heated tungsten wire as an infrared light source to measure the rough surface of large astronomical telescope mirrors. The primary advantage of a heated tungsten wire is its ability to be suspended in air and heated to high temperatures, producing high-contrast patterns in the infrared spectrum. However, tungsten wires are prone to low-ordered bending, making it difficult to maintain their shape during heating. Over time, changes in the power and emissivity of the tungsten wire further contribute to instability in the output light, which directly impacts the accuracy of surface reconstruction. In 2019, Graves et al. [17] designed a time-modulated long-wave infrared integrating cavity light source, incorporating a highly stable resistance film blackbody element within a heating cavity. The light was emitted through a mechanical slit. This design offered several advantages. The geometry of the light source was stable, the time-modulated signals allowed for effective signal extraction and background noise isolation, and the linear light source output—after multiple reflections within the cavity—was more uniform and stable. Compared to a heated tungsten wire, this method provided higher contrast signals and better shape stability. However, as the power of the light source increased, the number of required resistance films grew, leading to larger light source components and a proportional rise in cost. To address these limitations, this paper proposes a novel slit light source design based on a cavity structure using a heated polyimide film.

## 2. Measurement Principle of Infrared Deflectometry

**Figure 1:**
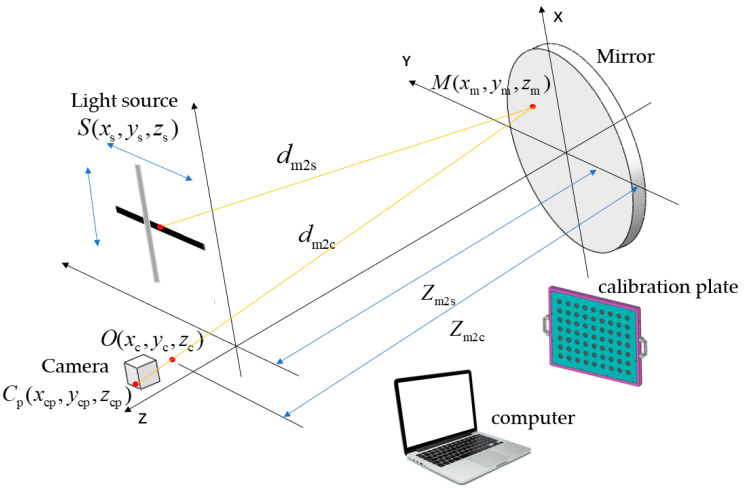
Measurement principle diagram of infrared deflectometry.

Deflectometry relies on the law of reflection in optics. The measurement principle of infrared deflectometry is illustrated in Figure 1.

The light source encodes a pattern in infrared light, which is then distorted upon reflection by the mirror surface. The camera captures these distorted fringe patterns. According to the law of reflection, the angular bisector of the incident and reflected light rays corresponds to the normal vector at the measured point on the mirror. Then, the surface gradient can be calculated accordingly, as shown in Equation (1), and the surface form can be reconstructed through the integration of surface slopes [18,19,20].

(1) $$\begin{array}{l} w_{x}\left(x_{m},y_{m}\right)=\frac{\frac{x_{m}\;-\;x_{s}}{d_{m2s}}+\frac{x_{m}\;-\;x_{c}}{d_{m2c}}}{\frac{z_{m2s}\;-\;W(x_{m},y_{m})}{d_{m2s}}+\frac{z_{m2c}\;-\;W(x_{m},y_{m})}{d_{m2c}}}\\ w_{y}\left(x_{m},y_{m}\right)=\frac{\frac{y_{m}\;-\;y_{s}}{d_{m2s}}+\frac{y_{m}\;-\;y_{c}}{d_{m2c}}}{\frac{z_{m2s}\;-\;W(x_{m},y_{m})}{d_{m2s}}+\frac{z_{m2c}\;-\;W(x_{m},y_{m})}{d_{m2c}}} \end{array}$$

where $x_{m}$ and $y_{m}$ are the coordinates of the measured point, $x_{c}$ and $y_{c}$ are the coordinates of the camera’s optical center, $x_{s}$ and $y_{s}$ are the coordinates of the corresponding light source, $z_{s}$ and $z_{c}$ are the *z*-coordinates of the light source and camera pixel, respectively, $d_{m2s}$ and $d_{m2c}$ are the spatial distances from the measured point to the light source and from the measured point to the camera center, respectively, $W(x_{m},y_{m})$ is the height of the measured point, and $w_{x}(x_{m},y_{m})$ and $w_{y}(x_{m},y_{m})$ are the slopes of the measured point in the *x* and *y* directions, respectively.

This requires that the measured mirror exhibit specular reflection. For visible light, a polished smooth surface can be considered as a specular reflector. However, for rough surfaces, where the surface roughness is larger, diffuse reflection of light occurs [21]. The main factors influencing the specular reflection of the surface can be described using the Rayleigh criterion [22], as expressed by

(2) $$R_{q}<\lambda /(8\cdot \cos\theta ),$$

where *λ* is the wavelength, *R_q_* is the surface roughness, and *θ* is the incidence angle. For visible light deflectometry, the wavelength is approximately 500 nm. If the incident light is normal to the surface, the roughness must be less than 60 nm to satisfy the Rayleigh criterion, which represents the current measurement limit in visible light deflectometry in terms of reflectivity. In the long-wave infrared range, the wavelength is around 10 µm, and the surface roughness must be less than 1.25 µm to meet the conditions for specular reflection. Therefore, by using light in the 8 µm to 14 µm range, rough surfaces can be measured more effectively with longer wavelengths.

**Figure 2:**
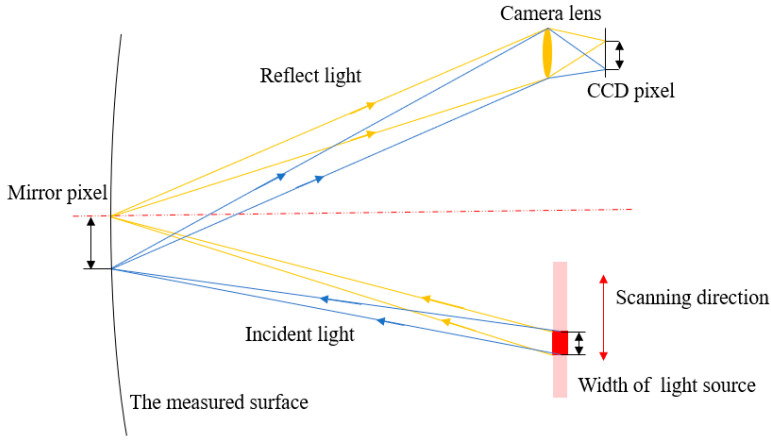
Schematic of infrared scanning technique.

According to the measurement principle, a single light source pixel is activated to illuminate a specific area of the measured surface, referred to as a “mirror pixel”. This illuminated area is then captured by a camera CCD. By repeating this process, the light source can sequentially illuminate and cover the entire mirror surface, as shown in Figure 2.

In the measurement system, the light source is an extended source of a certain size, and the pupil is not a perfect pinhole camera but also has certain dimensions. This makes it difficult to establish a direct relationship between the light source, the mirror, and the camera image pixels. The light source scans at different positions to illuminate different portions of the mirror surface, and the resulting light eventually converges onto the camera detector. As a result, a centroid calculation algorithm is required to determine the position of the light source. Let $x_{s}$ and $y_{s}$ represent the coordinates of the light source in the *x* and *y* directions, which can be calculated as

(3) $$\left\{\begin{array}{c} x_{s}={\sum x_{i}I}_{i}/{\sum I}_{i}\\ y_{s}={\sum y_{i}I}_{i}/{\sum I}_{i} \end{array}\right.,$$

where $x_{i}$ and $y_{i}$ are the coordinates of the light source in the *i*-th step of the scanning process, and $I_{i}$ is the gray value of the camera pixel. The average slope of a mirror pixel can be calculated using the centroid of multiple pixel values, which improves the accuracy of the mirror slope estimation. Noise in $I_{i}$ can degrade measurement accuracy. Therefore, an ideal infrared light source should exhibit good stability, uniformity in geometric structure, and a high signal-to-noise ratio.

## 3. Design and Calibration of the Infrared Light Source

### 3.1. Design of the Infrared Slit Light Source

**Figure 3:**
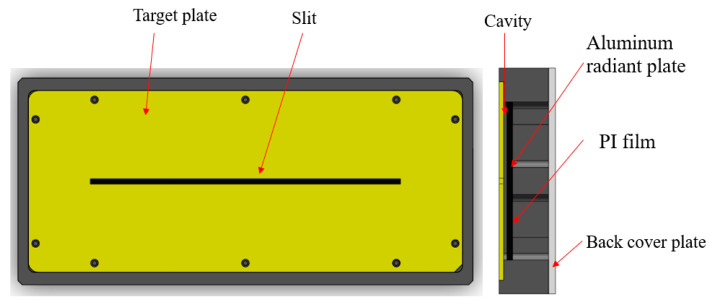
Structure of the infrared slit light source.

A slit light source design based on a heated polyimide (PI) cavity structure is proposed, as illustrated in Figure 3. The outer dimensions of the light source structure are 220 mm × 100 mm × 22 mm, and the air gap between the radiation plate and the target plate is 1.5 mm.

The infrared radiation source is a black aluminum plate uniformly heated by a PI film. PI film is a yellow-transparent, flexible metal-encapsulated electric thermal film with a sandwich structure. The upper and lower insulation layers are made of PI, which offers high insulation strength and excellent heat conduction efficiency. The heating element is constructed from a precision resistance alloy that can operate at temperatures up to 150 °C continuously and up to 250 °C for short durations. It is evenly attached to the back of the radiation aluminum plate to ensure uniform heating. The polyimide plate, with a precisely machined rectangular slit (150 mm × 2.5 mm), serves as a target plate. The straightness of the slit can achieve an accuracy of 0.01 mm. The radiation plate and target plate are separated by a polyimide material structure, forming a cavity that serves the thermal insulating function.

When the infrared light source is powered on, the infrared image shows the slit as a bright target against a uniformly dark background. The size of the slit can be adjusted by replacing the target plate, and both the outer frame and slit size can be customized according to suit specific requirements. Since the heating plate is exposed, a back cover plate is designed to provide both safety protection and thermal stability. The heating film cable is routed through a slot in the side wall of the outer frame. The output voltage from the adapter is converted into heating power for the PI film. An adjustable temperature controller is installed between the PI film and the adapter, with a temperature range from −50 °C to 200 °C. When the temperature reaches the pre-set value, the heated PI stops heating, protecting the element from being damaged. This controller also allows for flexible regulation of the radiation plate’s temperature, enabling the creation of slit targets with varying contrasts. The system is safe and convenient. For applications requiring significant temperature changes, a high-power DC regulated power supply can be used to achieve the desired results.

**Figure 4:**
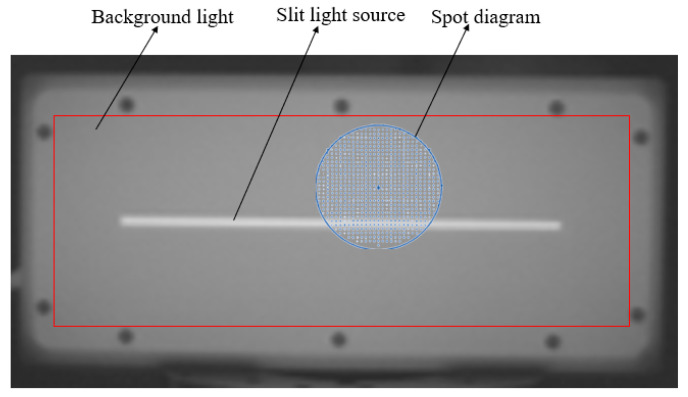
Measurement setup for the heated film slit light source.

In the long-wave infrared range, all objects at room temperature act as radiation sources, resulting in numerous noise sources within the test environment. This noise can degrade the signal-to-noise ratio and reduce measurement accuracy. As a result, software simulations, combined with the actual optical path distance, are used to calculate the size of the image field. The ray tracing is carried out in reverse, then the light emitted by the camera is reflected by the measured mirror and falls in the blue area of the light source target plate. During the scanning process, the diameter of the spot diagram (represented by the blue area) must not exceed the red border, which corresponds to about half the size of the target plate, as shown in Figure 4. This design allows the slit signal to be extracted from a uniform background radiation, producing a stable heat source signal with significant contrast. It effectively minimizes the impact of stray light on the measurement system, thereby improving the accuracy of the centroid calculation.

### 3.2. Reliability Verification of the Infrared Light Source

**Figure 5:**
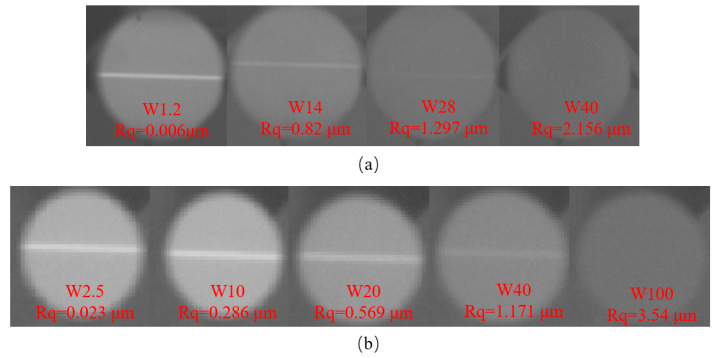
Comparison of infrared light reflection from surfaces with different roughness: (**a**) fused silica mirrors; (**b**) silicon carbide mirrors.

Silicon carbide and silica materials, known for their excellent optical properties, are widely used for fabricating large-sized components. To verify the reliability of the infrared slit light source based on heated PI film, an infrared camera was used to capture the thermal radiation signal reflected from the mirror’s surface, clearly imaging the slit. In Figure 5, the particle sizes of grinding abrasives and the corresponding surface roughness *Rq* of the mirrors under test are denoted in red. The designed slit light source based on heated PI film can generate thermal contrast patterns. After the slit light source is reflected by the mirror, the camera captures an image containing information about the measured mirror; the greater the roughness, the weaker the reflected light. When the roughness is approximately 1.25 µm, the reflected light is expected to vanish, and the infrared camera is unable to capture the signal. This not only validates the relationship between reflectance and roughness described in Equation (2) but also demonstrates that the light source design method proposed in this paper can be used in infrared deflectometry.

The infrared camera calibration plate is typically made from materials with different emissivities, creating a checkerboard or circular pattern to generate temperature differences that help identify image edges. A key advantage of the light source design proposed in this paper is its ability to enable infrared camera calibration [23,24] by replacing the slit on the target plate with a circular hole array.

### 3.3. Pre-Calibration of the Infrared Light Source Using an Optical Theodolite

**Figure 6:**
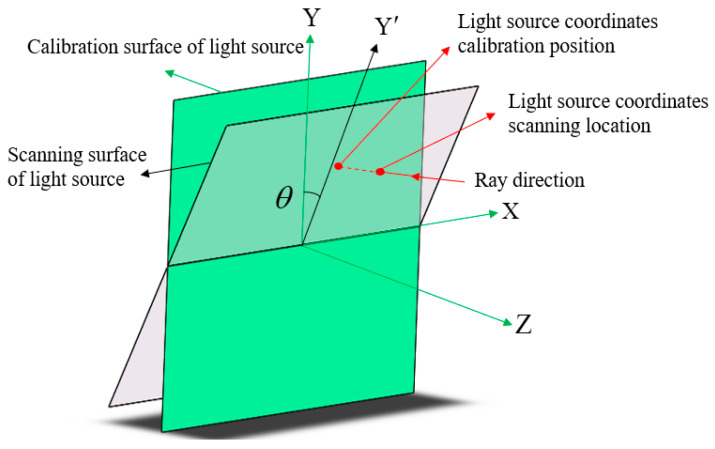
Schematic of the angular deviation between the scanning surface and the calibration surface of the infrared light source.

A mechanical adjustment frame is required to enable movement scanning of the slit in both the X and Y directions. During the scanning process, an angle exists between the slit scanning surface and the moving track surface of the guide rail, resulting in an angular error between the actual scanning surface of the light source and the calibration surface. The analysis of the tilt is shown in Figure 6. As detailed in [25], surface shape errors caused by angular errors are thoroughly analyzed. Tilt angle θ can be controlled to within 5”, and the resulting form error can be reduced to nanometer-level accuracy, which can be calculated via simulations.

When the light source is scanned, the guide rail moves from point A to point B. In practice, the light source does not follow the guide rail accurately, leading to a deviation in the actual movement distance. Without calibration, the calculated scanning position of the light source will move from point C to point E, whereas it should move from point C to point D.

**Figure 7:**
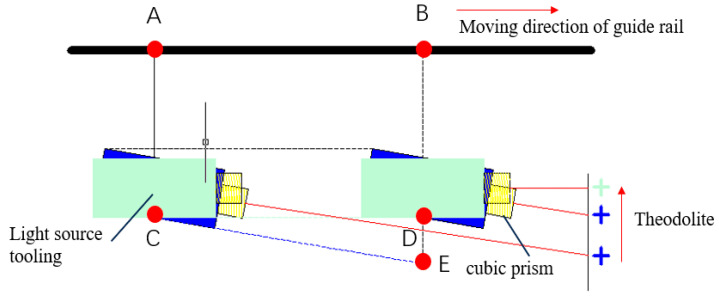
Schematic of calibrating the light source.

The schematic for the pre-calibration of the light source position is shown in Figure 7.

A cubic prism with a crosshair is attached at the side of the light source housing. Adjust the azimuth and elevation of the theodolite so that the returned image of the cube prism appears at the center of the theodolite’s field of view. As the guide rail moves, the cross pattern on the prism, such as the blue cross in Figure 6, will shift from left to right, indicating an angular deviation between the calibration surface of the light source and the guide rail direction. By adjusting the position of the light source, the cross in the center of the viewing prism on the theodolite can be made to remain stationary, as shown by the green cross in Figure 6. At this point, the calibration surface of the light source moves in sync with the guide rail, thus ensuring that the light source position is correctly calibrated.

## 4. Experimental Demonstration

A concave parabolic aluminum mirror with a diameter of 110 mm and a vertex curvature radius of 800 mm is adopted for demonstration purposes. The infrared light source follows the proposed design method, with a slit size of 150 mm × 2.5 mm. The light source is heated to 60 °C, and a DC power adapter with an adjustable output voltage range of 3–24 V and a power rating of 120 W is used as the power supply.

The adjustable frame includes both three-dimensional translational adjustment and 360-degree rotational adjustment capabilities. The XYZ single-axis repeat positioning accuracy is 1 µm, with rotational fluctuation of 15 µm and repeat positioning accuracy of 0.001°. This frame can achieve high-precision scanning transversely and can rotate 90° to switch the slit direction. The infrared camera used is an uncooled Gobi-640-GigE infrared camera, with its parameters listed in Table 1. The calibration plate for the measurement system is 190 mm × 170 mm, which contains 80 circular holes arranged in an 8 × 10 matrix. The spacing between adjacent holes is 15 mm. Among them, 78 holes have a diameter of 6 mm. To distinguish the orientation of the calibration board, two special holes with diameters of 4 mm and 8 mm are machined in the center as markers. The positional accuracy of the holes is ±0.01 mm.

**Figure 8:**
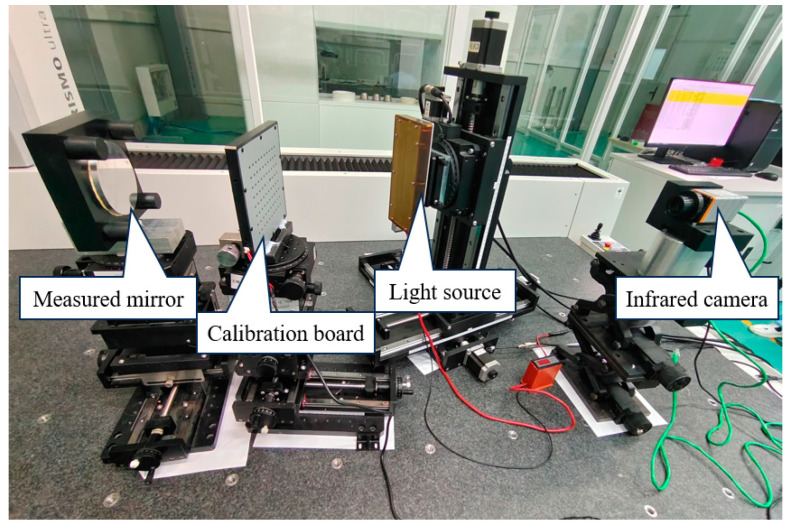
Experimental setup.

The optical measurement setup is shown in Figure 8.

### 4.1. Measurement Procedure

**Figure 9:**
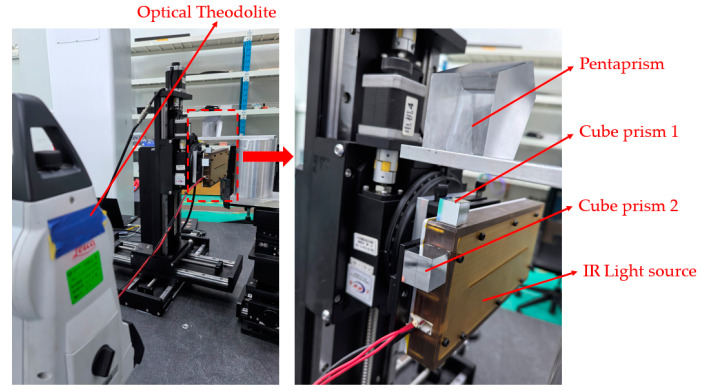
Setup of calibrating the light source.

Next, we attach a cubic prism marked with a crosshair to the left side and the top of the slit light source frame, as shown in Figure 9. We align the theodolite with the left plane of the cubic prism 2, ensuring that the reflected image of the plane is centered in the field of view. We continuously adjust the position of the slit light source structure. As the rail moves back and forth along the x-axis, the theodolite observes the finite distance position on the surface of the prism, ensuring that the crosshair at the center of the prism surface does not move during measurement. At this point, the slit light source is aligned with the scanning plane and the calibration plane along the x-axis, with an angular error of approximately 2 to 5 arcseconds between them. In the y direction, the rail moves up and down. Since the theodolite cannot directly measure the reflected image of the top surface of cubic prism 1, a pentaprism is placed on a perforated aluminum plate to deflect the light path, and then the theodolite is used to measure cubic prism 1. The calibration method is the same as that for the x axis.

After calibration, the angular difference between the light source and the guide rail is reduced to less than 5 arcseconds. The optical path setup is constructed on a CMM, and the relative positions of each component are adjusted so that the camera can clearly capture the pattern reflected by the mirror. This ensures that the light source’s target plate covers the full aperture of the mirror during the scanning process.

**Figure 10:**
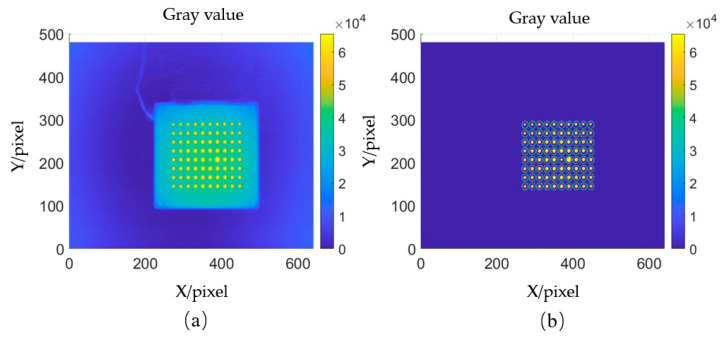
Reflected light calibration data processing method: (**a**) acquisition of calibration plate pattern; (**b**) calculation of circular spot centroid coordinates.

In this experiment, a model-free camera calibration method is used. The calibration plate is placed between the measured mirror and the camera, positioning it such that the light from the camera incident on the measured mirror covers the circular spot area of the calibration plate. Two images of the calibration plate are captured at different positions. One of the captured patterns from the calibration plate is shown in Figure 10a. The centroid coordinates of each circular spot are then calculated, as shown in Figure 10b. Using an interpolation algorithm, the coordinates of the two images corresponding to each pixel are determined, and a straight line is fitted through the two points to solve for the light direction corresponding to each camera pixel [26].

**Figure 11:**
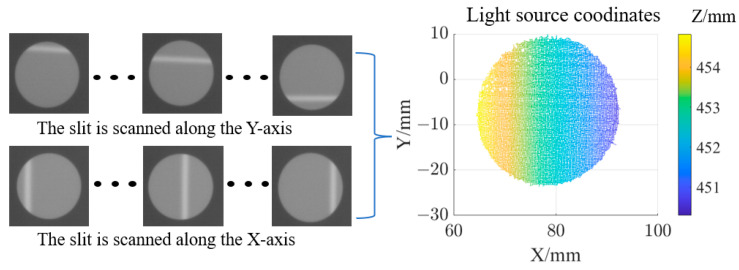
Calculation of light source coordinates.

The temperature of the light source is set to 60 °C, with a scanning interval of 0.25 mm in both the x and y directions. At each scanning position, the camera captures an image, and the centroid solving algorithm is applied to calculate the coordinates of the light source, as shown in Figure 11.

The position coordinates of the light source center, the measured mirror, and the calibration plate are determined using a CMM. The coordinate system is then converted and unified through the method of aluminum block marking on the mirror’s tooling [26]. The centroid solution method establishes a one-to-one correspondence between the camera pixels and the light source coordinates. By determining the intersection of the reflected light with the theoretical surface shape of the measured mirror, the coordinates of the measured points can be obtained. The Southwell method is then used to reconstruct the surface shape errors.

### 4.2. Analysis of Measurement Results

**Figure 12:**
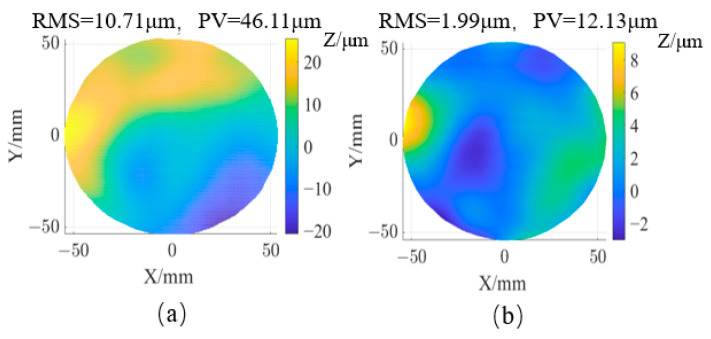
IRDM results: (**a**) without light source calibration; (**b**) with light source calibration.

**Figure 13:**
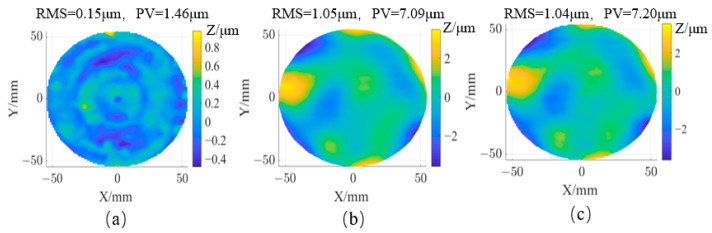
Result comparison: (**a**) reference result by CMM; (**b**) IRDM; (**c**) Deviation map with CMM.

Our analysis of the experimental results is presented in Figure 12 and Figure 13. In Figure 12a,b, the reconstruction results of IRDM are shown, both without light source calibration and after light source calibration. Using the same hardware setup and system calibration method, the experimental results indicate that the PV (Peak-to-valley) value of the surface error before and after light source calibration is 46.11 µm and 12.13 µm, respectively. This shows an improvement in reconstruction accuracy by approximately four times. The data above demonstrate that the structure and calibration method of the infrared slit light source are effective and can significantly enhance measurement accuracy.

In the field of optical processing, low-order aberrations can be adjusted [27]. Figure 13 compares the results after removing low-order aberrations (Z1–Z9). After light source calibration, the PV value and RMS of IRDM are 7.09 µm and 1.05 µm, respectively. After low-order aberration removal, the PV value and RMS of CMM are 1.46 µm and 0.15 µm. Figure 13c shows the pixel-by-pixel residual error (subtraction) between the IRDM and CMM data, indicating that IRDM can achieve 1 µm RMS measurement accuracy. The infrared light source design and calibration scheme proposed in this paper meet the measurement accuracy requirements for mirrors in the grinding stage, with the added advantage of significantly reducing measurement time.

The measurement light path of IRDM is set up in the processing environment, enabling in situ measurement during the manufacturing process. Only a single calibration is required to enable rapid measurement of optical mirrors at each processing stage, facilitating the fast iterative cycle of manufacturing–measurement–manufacturing and significantly improving measurement efficiency. In contrast, current CMM systems cannot perform in situ measurements, and laser trackers or measuring heads mounted on machines can only achieve a PV accuracy of around 30 µm. The infrared slit light source design and calibration method are inherently scalable and adaptable to a wide range of mirror geometries, including aspheric mirrors with large-apertures and large curvatures. The method employs a flexible slit light source, whose size and shape can be easily adjusted to accommodate different mirror geometries. For large-aperture mirrors, the slit size can be scaled up to ensure sufficient coverage of the mirror surface. Additionally, the method measures the slope variations on the mirror surface, making it inherently suitable for complex curvatures. For mirrors with a large curvature, measurement can be achieved by simply adjusting the distance of the measurement optical path and scaling the size of the calibration plate.

The infrared light source structure using PI heating films adopted in this paper only costs tens of yuan. However, the heating resistors used in Graves’ design cost about 500 yuan each, and the total cost is as high as tens of thousands of yuan. This significant cost difference makes our design more economically feasible for large-scale applications. Compared with the integrating cavity light source by Graves [17] and the heated tungsten ribbon light source proposed by Su et al. [15,16], this design has a regular geometric structure, which is convenient for calibration and alignment. As a result, it can improve the measurement accuracy of the low-order aberrations of the mirror and can generate uniform background radiation, which helps to eliminate the influence of background noise on the surface measurement accuracy. Through the above experimental verification, the light source structure designed in this paper has been shown to have significant advantages in terms of cost, performance, and practicality and has demonstrated its important engineering value in practical applications.

## 5. Conclusions

The novel design of an infrared slit light source based on heated PI has remarkable advantages, including geometric stability, high contrast, and ease of calibration. The deflectometric measurement system can effectively measure the form errors of rough surfaces with a roughness below 1 µm Sq, typically fabricated by grinding or finish milling. Infrared deflectometry offers low hardware costs, simple operation, and reliable results, especially for in situ measurements embedded into grinding machines, providing useful guidance for optical machining. Additionally, the repeated movement and alignment between the fabricating machine and measurement instrument are avoided, thereby significantly shortening the measurement period of meter-sized large optical components.

## Figures and Tables

**Table 1:** Camera specifications.

Items	Properties
Camera model	Gobi-640-GigE
Spectral band	8 μm~14 μm
Camera resolution	640 × 480
Pixel pitch	17 μm
Focal length	17 mm
Field of view Angle	±15°

## Data Availability

Data underlying the results presented in this paper are not publicly available at this time but may be obtained from the authors upon reasonable request.
